# Correction to “A Novel Fluorine Mass Balance Method for Improved Characterization and Quantification of Extractable (Organo)fluorine in Drinking Water”

**DOI:** 10.1021/acs.estlett.5c00233

**Published:** 2025-03-17

**Authors:** Zongzhe He, Merle Plassmann, Ian T. Cousins, Jonathan P. Benskin

We would like to make the following clarifications to our recently published article:^1^

(1) In the section “FMB in Stockholm Tap Water”, the stated units (ng/L) should have been “ng F/L” to emphasize that the concentrations are presented as fluorine equivalents.
(2) The molecular PFAS concentrations (i.e., units of ng/L) presented in Table S7 and Figure S3 can be converted to fluorine equivalent concentrations (ng F/L) using the weight percentage of fluorine in each substance. For the reader’s convenience, we provide here a comparison of the concentrations in units of both ng/L and ng F/L (Table 1).

**Table 1:** Average Concentrations and Standard Deviations (SD) of Fluorinated Substances in Stockholm Drinking Water^a^

**Compounds**	**Concentration** (ng/L)	**SD** (ng/L)	**Concentration** (ng F/L)	**SD** (ng F/L)
TFA	330.4	12.76	166.6	6.438
PFPrA	5.113	0.531	2.980	0.310
PFBA	3.703	0.447	2.312	0.279
PFPeA	3.083	0.168	2.005	0.109
PFHxA	1.561	0.072	1.042	0.048
PFHpA	1.182	0.093	0.804	0.063
PFOA	0.870	0.043	0.600	0.030
PFNA	0.284	0.023	0.198	0.016
TFMS	1.562	0.044	0.597	0.017
PFPrS	0.144	0.005	0.077	0.003
PFBS	0.945	0.071	0.541	0.041
PFPeS	0.197	0.016	0.118	0.010
PFHxS	0.879	0.025	0.544	0.015
PFHpS	0.086	0.022	0.055	0.014
PFOS	2.668	0.210	1.727	0.136
6:2 FTS	0.167	0.042	0.097	0.024
BF_4_^–^	8.692	0.551	7.610	0.482
PF_6_^–^	0.664^b^	0.170	0.522	0.134

a Both molecular (i.e. ng/L) and fluorine equivalent (i.e. ng F/L) concentrations are shown.

b The concentration of PF_6_^–^ was below the limit of quantification (1.726 ng/L) but above the limit of detection (0.440 ng/L).

This correction has no impact on the conclusions of the manuscript.
